# Dopamine elevates and lowers astroglial Ca^2+^ through distinct pathways depending on local synaptic circuitry

**DOI:** 10.1002/glia.23103

**Published:** 2016-11-29

**Authors:** Alistair Jennings, Olga Tyurikova, Lucie Bard, Kaiyu Zheng, Alexey Semyanov, Christian Henneberger, Dmitri A. Rusakov

**Affiliations:** ^1^ UCL Institute of Neurology, University College LondonLondonUnited Kingdom; ^2^ Institute of Neuroscience, University of Nizhny Novgorod603950 Nizhny NovgorodRussia; ^3^ RIKEN Brain Science InstituteWakoSaitamaJapan; ^4^ Institute of Cellular Neurosciences, University of Bonn Medical SchoolGermany; ^5^ German Center of Neurodegenerative Diseases (DZNE)BonnGermany

**Keywords:** astroglia, dopamine, calcium, synaptic transmission, hippocampus

## Abstract

Whilst astrocytes in culture invariably respond to dopamine with cytosolic Ca^2+^ rises, the dopamine sensitivity of astroglia *in situ* and its physiological roles remain unknown. To minimize effects of experimental manipulations on astroglial physiology, here we monitored Ca^2+^ in cells connected via gap junctions to astrocytes loaded whole‐cell with cytosolic indicators in area CA1 of acute hippocampal slices. Aiming at high sensitivity of [Ca^2+^] measurements, we also employed life‐time imaging of the Ca^2+^ indicator Oregon Green BAPTA‐1. We found that dopamine triggered a dose‐dependent, bidirectional Ca^2+^ response in *stratum radiatum* astroglia, a jagged elevation accompanied and followed by below‐baseline decreases. The elevation depended on D1/D2 receptors and engaged intracellular Ca^2+^ storage and removal whereas the dopamine‐induced [Ca^2+^] decrease involved D2 receptors only and was sensitive to Ca^2+^ channel blockade. In contrast, the *stratum lacunosum moleculare* astroglia generated higher‐threshold dopamine‐induced Ca^2+^ responses which did not depend on dopamine receptors and were uncoupled from the prominent inhibitory action of dopamine on local perforant path synapses. Our findings thus suggest that a single neurotransmitter—dopamine—could either elevate or decrease astrocyte [Ca^2+^] depending on the receptors involved, that such actions are specific to the regional neural circuitry and that they may be causally uncoupled from dopamine actions on local synapses. The results also indicate that [Ca^2+^] elevations commonly detected in astroglia can represent the variety of distinct mechanisms acting on the microscopic scale. GLIA 2017;65:447–459

**Abbreviations**
AC: Adenylyl cyclase
cAMP: Cyclic adenosine monophosphate
DA: Dopamine
FLIM: Fluorescence life‐time imaging
GJC: Gap‐junction connected
KMS: Potassium methyl‐sulphonate
PAC: Patched astrocyte cells
PKA: Protein kinase A
PLC: Phospholipase C

## Introduction

Dopamine (DA) is a neuromodulator which exerts powerful behavioral effects and thus has been implicated in numerous psychiatric conditions. However, the exact mechanisms by which DA acts on brain circuits remain poorly understood, partly because of the exceedingly wide range of its effectors (Bjorklund and Dunnett, 2007; Tritsch and Sabatini, 2012; Vaarmann et al., 2010). The primary signaling targets of DA are D1‐ and D2‐like receptors which trigger somewhat distinct signaling pathways (reviewed in (Beaulieu and Gainetdinov, 2011)). In brief, D1‐like receptors boost the production of cyclic adenosine monophosphate (cAMP) and activate protein kinase A (PKA), by acting upon G proteins Gα(s) and Gα(olf) and thus stimulating adenylyl cyclase (AC). D2‐like receptors, in contrast, activate Gα(i) and Gα(o) proteins thus inhibiting AC and suppressing PKA activation. Another important DA signaling channel involves Gα(i/o) coupled receptors (of the D2‐like subtype) which could act by freeing the Gβγ subunit. The latter can regulate ion channels controlling neuronal excitability, such as the inward‐rectifier K^+^ channels (Beckstead et al., 2004) or N‐ and L‐type Ca^2+^ channels modified through activation of phospholipase C (PLC). Among the prominent downstream actions of DA on neurons are the altered NMDA receptor sensitivity (Snyder et al., 1998) and induced intracellular Ca^2+^ waves (Surmeier et al., 1995). Intriguingly, in the hippocampus, DA has been found to modulate memory formation (da Silva et al., 2012; Gasbarri et al., 1996; Li et al., 2003) and to profoundly inhibit the perforant path input to CA1 (Otmakhova and Lisman, 2000) while also boosting long‐term synaptic potentiation, with little effect on basal transmission, at Schaffer collateral synapses (Otmakhova and Lisman, 1996; Otmakhova and Lisman, 2000).

There has also been evidence for DA actions in cultured astroglia. D1‐type receptors have been found in cortical astrocytes *in vitro* (Requardt et al., 2012; Zanassi et al., 1999). Their activation stimulates cAMP production and PKA activation (Zanassi et al., 1999), boosts the expression of GDNF, NGF (Ohta et al., 2010), and FGF‐2 (Li et al., 2006), and modulates NADH and astroglial Ca^2+^ signaling (Requardt et al., 2012). Cultured astrocytes also express a newly identified, PI‐linked D1‐like receptor associated with Ca^2+^ mobilization (Liu et al., 2009b). Similarly, D2‐type receptor activation has been linked to increases in BDNF, GDNF and NGF mRNA expression and protein synthesis (Ohta et al., 2010), FGF‐2 secretion (Li et al., 2006), and reduction of S100β secretion (Nardin et al., 2011) in cultured astrocytes, as well as suppression of αβ‐crystallin mediated neuroinflammation *in vivo* (Shao et al., 2013). Also, it has long been known that in culture D2‐type receptor activation induces Ca^2+^ elevations in astrocytes (Khan et al., 2001; Reuss and Unsicker, 2001), a phenomenon with multiple functional facets (Khakh and Sofroniew, 2015; Verkhratsky, 2005). It has recently been shown that in certain conditions such elevations could be inhibited by monoamine oxidase B inhibition, suggesting the involvement of the ensuing DA breakdown and free‐radical generation (Vaarmann et al., 2010). Notably, with the exception of one study (Shao et al., 2013), the DA signaling mechanisms listed above have been investigated in cultured or isolated cells. Given the well‐documented major differences between astroglia in culture and those *in situ*, the degree of expression and the adaptive significance of such mechanisms in organized brain tissue are yet to be ascertained.

Astrocytes have increasingly been implicated in the modulation of the synaptic efficacy and its use‐dependent changes *in situ* and *in vivo* (reviewed in (Dityatev and Rusakov, 2011; Halassa and Haydon, 2010; Nedergaard and Verkhratsky, 2012; Pannasch and Rouach, 2013; Perea and Araque, 2010)). In most cases, such actions depend on astrocytic Ca^2+^ signals, which appear to provide an endogenous communication medium for these otherwise non‐excitable cells (recently reviewed in (Rusakov, 2015; Volterra et al., 2014)). Since DA has been reported to induce Ca^2+^ rises and associated molecular cascades in astroglial cultures (see above), the question arises whether such astrocytic signals could mediate effects of DA on local neural circuitry in organized tissue. We therefore set out first to establish whether astrocytes *in situ* respond to DA with an intracellular Ca^2+^ change—and if so, by what mechanism—and second to ascertain whether such a response could mediate previously reported DA‐induced modulation of excitatory synaptic transmission in the hippocampus.

## Materials and Methods

### Hippocampal Slice Preparation

All experiments were carried out in accordance with the national and international rules and regulations for animal experimentation including EU Directive 2010/63/EU of 22 September 2010. Acute hippocampal slices, 350 μm thick, were prepared from 3‐4‐week‐old male Sprague–Dawley rats. Animals were anaesthetized to death with a lethal intraperitoneal injection of Sodium Pentobarbitol. The skull was opened up and the brain excised into an ice‐cold slicing solution containing (in mM): NaCl 50, sucrose 105, KCl 2.5, MgCl_2_ 7, NaH_2_PO_4_ 1.25, CaCl_2_ 0.5, Ascorbic acid 1.3, Sodium Pyruvate 3, and glucose 6 (osmolarity 304–312 mOsM), continuously bubbled with 95% O_2_/5% CO_2_. The whole hippocampus was dissected, placed in an agar block and transverse slices were prepared using a Leica VT 1200S slicer. Slices were then transferred to slicing solution kept at 34°C for 15 min before being transferred to an interface or an immersion chamber containing Ringer solution comprised of (in mM): NaCl 119, KCl 2.5, MgSO_4_ 1.3, NaH_2_PO_4_ 1, NaHCO_3_ 26, CaCl_2_ 2, glucose 10 (osmolarity 298‐302 mOsM). Ringer was kept at room temperature, and continuously bubbled with 95% O_2_/5% CO_2_. Slices were rested in Ringer for at least 45 minutes before recording. All experimental protocols were carried out in full compliance with UK guidelines on animal experimentation. For recordings, slices were transferred to a submersion‐type recording chamber and perfused with Ringer solution saturated with 95%O_2_/5%CO_2_. Experiments were carried out at 32–33°C unless specified otherwise.

### AM‐Ester Dye Loading

For imaging astrocytes loaded with sulforhodamine101 (SR101) and Fluo‐4‐AM, slices were allowed to rest for 30 minutes in Ringer solution, then incubated in Ringer containing 10 μM SR101 for 10 minutes at 35°C. Slices were then washed three times in plain Ringer and then incubated in Ringer containing 5μM SR101, 5μM Fluo‐4‐AM and 0.04% pluronic acid for 40 minutes at 35°C. Slices were then washed three times in Ringer, rested in Ringer at room temperature for 30 minutes and were then ready for recording.

### Whole‐Cell Astrocyte Patch‐Clamp

Micropipettes, pulled from borosilicate, filamented glass, were used for astrocyte whole‐cell patch‐clamp and pipette resistances were 3‐5MΩ. The standard cell patch procedures in an acute slice were followed as described (Zheng et al., 2015). Intracellular solution was potassium methyl‐sulphonate (KMS) based solution containing (in mM): KCH3O3S 135, HEPES 10, disodium phosphocreatine 10, MgCl2 4, Na2ATP 4, NaGTP 0.4 (pH adjusted to 7.2 with KOH, osmolarity 290–295 mOsM). For cell imaging and [Ca^2+^] monitoring, two fluorescent dyes were added to the intracellular solution, Alexa Fluor 594 (50 μM) as a morphological marker and Fluo‐4 (200 μM) as a Ca^2+^ indicator. For every patched astrocyte, the membrane current‐voltage (I‐V) relationship was plotted by stepped injection of current through the patch pipette, at 200 pA steps. Positive electrophysiological identification of a passive protoplasmic astrocyte required a linear I‐V relationship and a low input resistance (<10 MΩ) as well as a resting membrane potential lower than −80 mV (Henneberger and Rusakov, 2012).

### Extracellular Field Recordings

Electrical signals were acquired through an Axon instruments CV‐7B headstage, connected to the pipette solution by a chlorided silver wire, and a Multiclamp 700B amplifier. Each primary output was connected to a Humbug to reduce 50Hz background noise. Signals were filtered at 3–6 kHz, digitized and sampled through an analogue‐to‐digital converter, either an Axon CNS 1440A, or National Instruments BNC 2090, at 10 KHz. Software used for acquisition was either WinCP 4.2.1 or Clampex 10.2. Excitatory post‐synaptic field potentials (fEPSPs) were recorded through glass micropipettes of 1‐2 MΩ resistance. For Schaffer collateral stimulation, axonal fibers were stimulated with a bipolar electrode from a Digitimer DS3 constant current stimulator box. The stimulating electrode was placed in *stratum radiatum*, 100‐200 μm closer to CA3 then the recording electrode. The configuration was allowed to settle for up to 10 minutes and then the stimulus intensity was gradually increased until no further increase in the fEPSP slope was seen. The stimulus power was then adjusted to give 50‐60% of the maximal fEPSP slope – stimulus power did not exceed 70 μA and lasted 100 μs. Paired‐pulse stimuli were given with an inter‐stimulus interval of 50 ms and repeated every 15 s.

### Two‐Photon Excitation Imaging

Two setups were used to record fluorescent intensity images; either a modified Bio‐Rad Radiance 2000 on an Olympus BW50 microscope with a 40x objective, or a Fluoview FV1000 MPE microscope with a 25x objective. Both were optically linked to a separate femtosecond pulse TiSapphire MaiTai laser (SpectraPhysics) set to emit at 800 nm. Laser power was kept between 5 and 8 mW for bulk‐loaded Fluo‐4‐AM imaging, 2–5 mW for patch‐pipette‐loaded Fluo‐4 fluorescence imaging (as measured under the objective at 800 nM). Alexa Fluor 594 normally equilibrated across the astrocyte tree within 10–15 min (Zheng et al., 2015). During recordings, images were acquired simultaneously as frame scans in the Alexa emission channel (red) (540LP/700SP filter) and the Fluo‐4 emission channel (green) (515LP/530SP filter). When recording from bulk‐loaded astrocytes unreliable dye loading of processes, and relatively poor signal to noise ratio (in comparison to whole‐cell patch‐clamp loaded astrocytes), necessitated data acquisition from somata alone. Image zoom was adjusted to best capture the salient features of the imaged cells—somata of GJCs and visible (within resolution) patched astrocyte processes. Gain for both channels was kept the same and constant within a particular 2‐P setup. Pixel dwell time was kept constant (4 μs). Images were acquired at 1Hz. If cells failed to show protoplasmic astrocytic morphology after 10–15 minutes dye equilibration and GJCs they were discarded. If cells showed abnormally high resting Fluo‐4 fluorescence signal compared to the morphological marker, they were likewise discarded.

### Recording From Gap‐Junction Coupled Astrocytes

This entails whole‐cell patch‐clamp of an astrocyte and the subsequent imaging of nearby gap‐junction connected (GJC) astrocytes. The procedure usually required low input resistance and ∼30 min for dye diffusion through the syncytium, the procedures similar to standard one‐cell patch‐clamp experiments. For these recordings a recording configuration was chosen to allow simultaneous recording from both the patched cell processes and the GJC somata. In a subset of experiments, only GJC somata could be recorded from. Recordings were only made when the diffusion of the dye from the pipette into the syncytium had equilibrated, as measured by the intensity of the reference dye, Alexa Fluor 594. If significant (>10%) fluctuations in Alexa fluorescence intensity in GJCs were witnessed, the recording was discarded.

### Image Analysis

Regions of interest (ROIs) were divided into four categories based on the morphology in the Alexa 594 channel: Patched astrocyte (PAC) soma; PAC process; and GJC soma. Only visible structures with clear astrocyte morphology were chosen as ROIs. An area of each image showing no fluorescence from the morphological marker was chosen to give a measure of average background fluorescence. For each ROI the average pixel fluorescence was taken for each frame and the average background fluorescence for that frame was subtracted (to correct for instrumental noise). In control trials and where indicated, Fluo‐4 signal (green, G) was normalized to the morphological marker signal (red, R) to correct for fluorescence changes due to tissue movement (giving G/R) and laser power fluctuation. Fluo‐4 signals presented here have been normalized to the average fluorescence from each ROI in the baseline phase of each recording, to give Δ*F*/*F*_0_.

### Monitoring Intracellular Ca^2+^ Concentration With Fluorescence Life‐Time Imaging (FLIM) of OGB‐1

These measurements were based on the well‐established OGB‐1 life‐time sensitivity to nanomolar concentrations of free Ca^2+^ (Wilms et al., 2006) and carried out using a two‐photon excitation Femto2D microscope equipped with FLIM detectors (Femtonics, Budapest) using optimised and calibrated readings of OGB‐1 life‐time sensitivity to [Ca^2+^], as detailed earlier (Zheng et al., 2015). Cell‐impermeable OGB‐1 was added to the astrocyte patch pipette solution at 200 µM. Other protocols pertinent to patch‐clamp and imaging were similar to those used in intensity measurements of Fluo‐4 fluorescence in GJC astroglia (above).

### Sampling and Statistics

Throughout our testing we normally used one slice per animal, and recorded 1–3 cells per slice. Because in such experiments the greatest source of variance are individual cells and because the effects in questions are documented as a real‐time change in individual cells (i.e., no inter‐cell or inter‐slice comparisons), individual cells are referred to as a statistical unit, to follow the practice of real‐time single‐cell physiology studies.

Physiological viability of imaged astrocytes was routinely checked by observing their spontaneous Ca^2+^ fluctuations. All recorded cells were routinely added to the statistical sample: the *a priori* rejection criterion was the baseline fluorescence approaching the microscope detection threshold (thus affecting *ΔF/F* measures) or being too weak for FLIM acquisition. Astrocytes showing any significant (>20%) experiment‐wise trend in baseline fluorescence were also discarded. Samples were tested with the Shapiro‐Wilks test for normality; for comparing populations with a normal distribution, the Student's *t*‐test was used, otherwise, the Mann‐Whitney test was used.

## Results

### Dopamine Induces Prominent Ca^2+^ Responses

**Figure 1:**
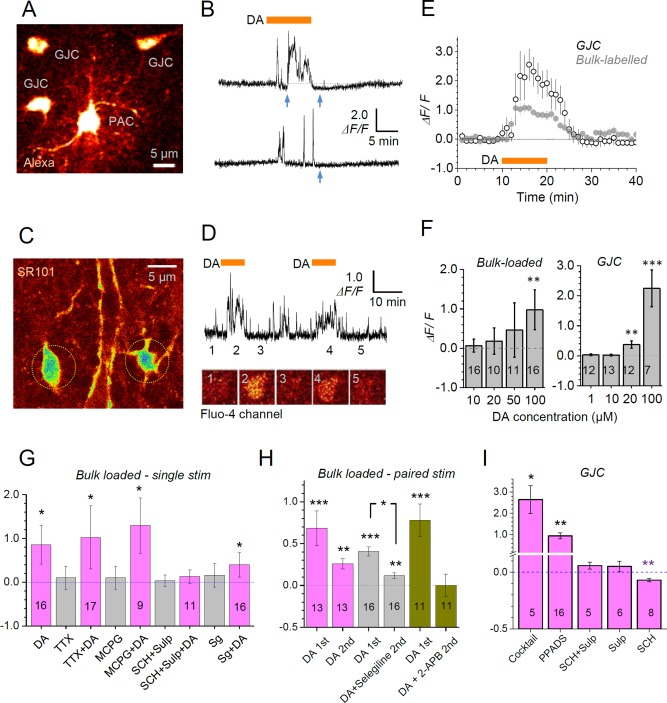
Dopamine‐triggered Ca^2+^ responses in CA1 *stratum radiatum* astroglia. **A**: Example of a patched (PAC) and GJC astroglia (Alexa 594, λ_x_^2P^ = 800 nm). **B**: Traces, characteristic Ca^2+^‐dependent fluorescence (Fluo‐4 channel) monitored in GJC astroglia (soma) during application of dopamine (100 µM, orange bar); blue arrows, clear negative defections of Ca^2+^ signal indicating decreases in [Ca^2+^]. **C**: Example of astroglia bulk‐labeled with sulforhodamine 101 (SR101) and Fluo‐4 AM (SR101 channel, *λ*_x_^2P^ = 800 nm). **D**: Traces, characteristic Ca^2+^‐dependent fluorescence (Fluo‐4 channel) monitored in bulk‐labeled astroglia (soma) during paired bath application of dopamine (100 µM, grey bars); image panels, illustration of Fluo‐4 channel recording of individual astroglia corresponding to experimental epochs 1‐5, as in the traces. **E**: Average time course (mean ± SEM) of Ca^2+^‐dependent fluorescence (Fluo‐4 channel) in GJC (open circles, *n* = 16) and Fluo‐4 AM bulk‐labeled astroglia (grey circles, *n* = 14), as indicated, in response to application of dopamine (100 µM, orange bar; single‐stimulus DA responses only). **F**: Dose dependence of dopamine‐induced astroglial Ca^2+^ response (ΔF/F, mean ± SEM amplitude) in bulk labeled and GJC astroglia, as indicated; digits inside columns, sample size; **P < 0.01, ***P < 0.005. G: Average amplitude (*ΔF/F*, mean ± SEM) of Ca^2+^ response to dopamine (application as in B) in bulk‐loaded astroglia in the presence of TTX (1 μM; responses to drug application alone, before DA application are shown in grey here), non‐specific mGluR blocker MCPG (200 µM), D1/5 receptor blocker SCH23390 (20 μM) and D2/3 receptor blocker sulpiride (50 μM) (SCH+Sulp), and monoamine oxidase B (MAOb) inhibitor selegiline (20 μM, Sg), as indicated; digits inside columns, sample size. *P < 0.05. H: Average amplitude (*ΔF/F*, mean ± SEM) of Ca^2+^ response in Fluo‐4 AM bulk‐loaded astroglia to paired‐application of dopamine (as in D), with the drug applied before the second stimulus: control, selegiline (20 μM), and IP3 receptor blocker 2‐APB (100 µM); digits inside columns, sample size; **P < 0.01, ***P < 0.005. I: Average amplitude (*ΔF/F*, mean ± SEM) of Ca^2+^ response in GJC astroglia to dopamine (100 µM, as in A‐B), in the presence of a drug cocktail containing TTX (1 μM), mGluR5 blocker MPEP (1 μM), mGluR5 blocker LY367385 (100 μM), mGluR2/3 blocker LY341495 (500 nM) and GABAB receptor blocker CGP (1 μM); a selective purinergic P2X antagonist PPADS (100 µM), D1/5 receptor blocker SCH23390 (20 μM) and D2/3 receptor blocker sulpiride (50 μM) (SCH+Sulp), sulpiride only (50 µM), and SCH23390 only (10 µM); *P < 0.05; **P < 0.01 (note two‐scale ordinate). [Color figure can be viewed at wileyonlinelibrary.com]

In the *stratum radiatum* of the hippocampal CA1 region, we held astrocytes in whole‐cell mode loading them with the morphological tracer Alexa 594 and Ca^2+^ indicator Fluo‐4, as detailed previously (Henneberger et al., 2010). Within 10‐15 minutes we were able to visualize the local astrocyte syncytium (Fig. 1A) including the patched astrocyte (PAC) and cells connected to the PAC via gap junctions (GJC cells), consistent with earlier reports (Giaume et al., 2010; Zheng et al., 2015). Bath‐applied DA (100 μM for 10 min, see controls for neuronal involvement below) had no detectable effect on Ca^2+^ in the PAC somata, possibly because of the proximity of the dialyzing pipette. However, it triggered robust [Ca^2+^] elevations in GJC somata (Fig. 1B).

To confirm this basic observation with an alternative imaging method, we labeled astroglia in bulk by slice incubation with cell‐permeable Fluo‐4 AM (Fig. 1C; astrocytes were identified in a 'red' emission channel using sulforhodamine 101, SR101). These bulk‐loaded cells displayed Ca^2+^ responses to DA which were qualitatively similar to those in GJC cells (Fig. 1D) although approximately half the size on average (Fig. 1E). To control for signal rundown and to minimize false‐negatives in pharmacological dissection trials, DA was applied twice in some of these experiments (Fig. 1D, see below). Dose‐response tests indicated that bulk‐loaded astroglia was substantially less sensitive than GJC cells to DA application (Fig. 1F). Overall, the Ca^2+^ responsiveness of astroglia to DA in acute brain slices was qualitatively consistent with that reported previously in cultured astroglia (Khan et al., 2001; Parpura and Haydon, 2000; Reuss and Unsicker, 2001; Vaarmann et al., 2010). Thus, we confirmed that Ca^2+^ responses documented here were most prominent in GJC cells which underwent minimal perturbation due to the pipette dialysis, and therefore were likely to preserve the endogenous mechanisms of Ca^2+^ homeostasis, as reported recently (Zheng et al., 2015). Intriguingly, in recordings from individual GJC astroglia we could clearly detect reductions in basal Ca^2+^ signal, upon and after DA application (Fig. 1B, blue arrows). These, however, were masked by the more prominent Ca^2+^ signal elevations when averaged across cells (Fig. 1E). In our subsequent experiments we therefore attempted (a) to dissect the basic molecular machineries responsible for [Ca^2+^] elevations and decreases, and (b) to validate these observations using the imaging method which is highly sensitive to nanomolar [Ca^2+^] but not to the possible concomitants of fluorescence intensity readout (such as cell volume changes or focus drift).

### DA‐Induced Ca^2+^ Elevations Rely on DA Receptors But Do Not Depend on Neuronal Spiking, Metabotropic Glutamate or GABA Receptors

Application of DA could potentially prompt changes in local neuronal activity, including previously documented changes in excitatory transmission (Otmakhova and Lisman, 2000). Nerve cell activity could in turn evoke astroglial Ca^2+^ transients, notably through metabotropic glutamate receptors or GABA_B_ receptors expressed in hippocampal astrocytes (Hamilton and Attwell, 2010). To dissect neuronal influences, we repeated our experiments in the presence of Na^+^‐channel blocker TTX (1 μM), mGluR5 blocker MPEP (1 μM), mGluR5 blocker LY367385 (100 μM), mGluR2/3 blocker LY341495 (500 nM) and GABA_B_ receptor blocker CGP (1 μM), separately or in a cocktail. This receptor blockade did not appear to affect DA‐induced Ca^2+^ elevations in either bulk‐loaded or GJC astroglia whereas such elevations were completely blocked by the mixture of the D1/5 receptor blocker SCH23390 (20 μM) and D2/3 receptor blocker sulpiride (50 μM), or alternatively by sulpiride alone (20 μM) (Fig. 1G‐I). The latter result has suggested that activation of D1/5 receptor on its own (or receptors beyond D1/5 and D2/3 types) is unable to induce detectable Ca^2+^ signals in the astroglia under study. In addition, a selective purinergic P2X antagonist PPADS (100 µM) appeared to halve the DA‐induced Ca^2+^ rise (Fig. 1I, difference 1.16 at *P* = 0.026 compared to control in Fig. 1E‐F) suggesting a role for ATP in boosting astroglial Ca^2+^ rises (Pascual et al., 2005).

Inhibition of monoamine oxidase B (MAOb) with selegiline (20 μM) has been reported to inhibit DA‐induced Ca^2+^ signaling in astrocytes through reactive oxygen species production (ROS) leading to lipid peroxidation and downstream PLC‐mediated activation of the IP3 pathway (Vaarmann et al., 2010). We found that the DA stimulus still produced a detectable Ca^2+^ response in the presence of selegiline although this response tended to be smaller than the one without selegiline (Fig. 1G,H). Thus, in CA1 astroglia *in situ* the MAOb cascade appears to play a less critical role in mediating the DA action if compared to that in cultured astrocytes (Vaarmann et al., 2010).

Intriguingly, Ca^2+^ imaging in GJC cells detected that the D1/5 receptor blockade with SCH23390, while abolishing DA‐induced [Ca^2+^] *elevations*, unmasked DA‐induced [Ca^2+^] *decreases* in these cells (Fig. 1I). As mentioned above, such decreases were apparent in individual cell recordings, during and after the DA stimulus (Fig. 1B, blue arrows). Because this uncommon observation relied on the fluorescence intensity measures and thus could be influenced, for instance, by small volume changes in recorded cells, we explored it further, as explained in the sections below. First, however, we sought to identify cellular mechanisms mediating the prominent Ca^2+^ elevations documented in the experiments above.

### DA‐Induced Ca^2+^ Increases Depend on Ca^2+^ Storage and Removal Mechanisms

**Figure 2:**
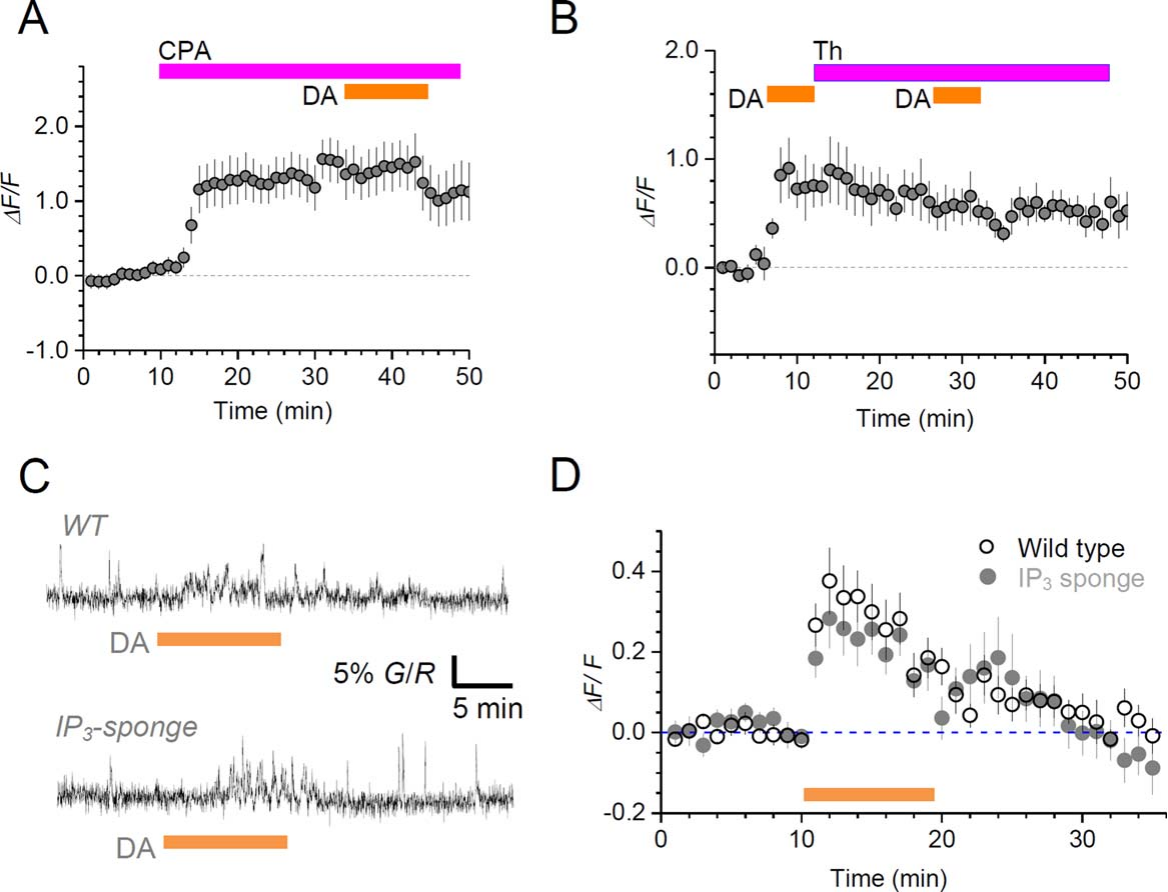
Dopamine‐induced astroglial Ca^2+^ elevations depend on intracellular Ca^2+^ storage and removal but not on IP_3_ diffusion buffering. **A**: Average time course (mean ± SEM, *n* = 9) of Ca^2+^ ‐dependent fluorescence (*ΔF/F*, Fluo‐4) in GJC astroglia during the application of cyclopiazonic acid (CPA) (30 µM) and, subsequently, DA (100 µM), as indicated. **B**: Average time course (mean ± SEM, *n* = 3) of Ca^2+^ ‐dependent fluorescence (*ΔF/F*, Fluo‐4) in bulk‐loaded astroglia during the application of DA (100 µM, 5 min), then thapsigargin (Th, 5 µM), and another DA application, as indicated. **C**: Characteristic Ca^2+^ responses (Fluo4 fluorescence) to DA application (100 μM, grey bar) in bulk‐loaded hippocampal astrocytes from wild‐type mice (WT, top) and IP_3_‐sponge mice (bottom). **D**: Average time course (mean ± SEM) of *ΔF/F* Ca^2+^ responses to DA (as in C) from wild‐type mice (hollow circles, *n* = 18) and IP_3_‐sponge mice (grey circles, *n* = 16). [Color figure can be viewed at wileyonlinelibrary.com]

It has long been known that IP_3_ receptor‐driven internal Ca^2+^ stores are a major source of activity‐induced intracellular Ca^2+^ elevation in astroglia (Fiacco et al., 2009; Volterra et al., 2014). First, we found that application of the IP_3_ receptor blocker 2‐APB (100 μM) completely abolished the DA‐evoked Ca^2+^ response in experiments with paired application of DA (to minimize false negatives; Fig. 1H). To explore the pathway of intracellular Ca^2+^ homeostasis further, we tested the effect of the Ca^2+^ pump inhibitor CPA (30 µM). Applied in bath, CPA induced a prominent Ca^2+^ elevation (consistent with the suppression of intracellular Ca^2+^ removal), which occluded any effects of DA applied subsequently (Fig. 2A). We next modified our test and applied a Ca^2+^ store inhibitor thapsigargin (5 µM) immediately after the first DA stimulus, to test whether the thapsigargin‐induced depletion and subsequent blockade of Ca^2+^ stores, as documented earlier in neuronal axons (Scott and Rusakov, 2006), would affect responses to a second DA stimulus. We found that thapsigargin maintained, but did not increase, the Ca^2+^ elevation produced by first DA stimulus while occluding any Ca^2+^ responses to the second DA stimulus (Fig. 2B). These results suggest therefore that intracellular mechanisms of Ca^2+^ storage (possibly IP_3_ receptor‐dependent) and removal play a major role in generating DA‐induced Ca^2+^ elevations in our experiments.

Finally, to find out whether such Ca^2+^ elevations depend on diffusion of the IP_3_ receptor ligand IP_3_ across the cell volume (as opposed to a highly localized IP_3_ action), we used a genetic animal model that produced an IP_3_ 'sponge' protein complex targeted to astroglia. This IP_3_ ‘sponge’ exhibits 1000‐fold greater affinity to IP_3_ than the native IP_3_(1) receptor (Uchiyama et al., 2002). It was shown previously that in the IP_3_‐sponge animals astrocytic Ca^2+^ waves evoked by the classical mGluR‐IP_3_ cascade were significantly attenuated, thus implicating intracellular IP_3_ diffusion as an important contributor to the wave generation or propagation (Tanaka et al., 2013). In contrast, in our experiments DA‐induced Ca^2+^ signals in the IP_3_‐sponge animals were indistinguishable from those in the wild type (Fig. 2C,D). These data suggest that, unlike mGluR agonists, DA engages IP_3_ receptors on a small scale, possibly within a nanodomain: at this scale, the effect of sponge‐like IP_3_ buffer is likely to be minimal, akin to what has long been demonstrated in studies of intracellular Ca^2+^ diffusion and buffering (Eggermann et al., 2012).

### DA Elevates and Lowers Astroglial Ca^2+^ Engaging Distinct DA Receptor Mechanisms

**Figure 3:**
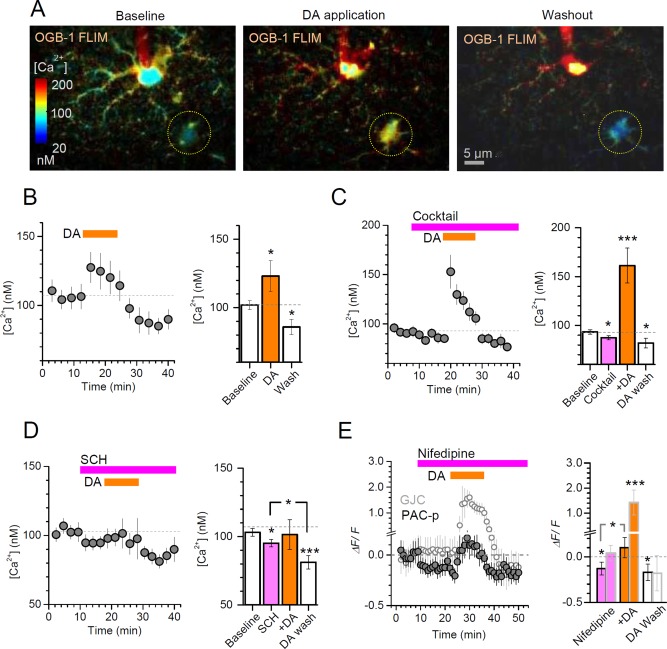
Dopamine evokes a bi‐phasic, bi‐directional Ca^2+^ response in CA1 astroglia. **A**: An example showing the monitoring of basal [Ca^2+^] in CA1 GJC astroglia (dotted circle) with two‐photon excitation FLIM of OGB‐1 (as in (Zheng et al., 2015)) loaded through the patched cell (centre); internal [Ca^2+^] levels are colour‐coded, as indicated; averaging window is 2 min. **B**: Plot, average time course of intracellular [Ca^2+^] (mean ± SEM, *n* = 8) during application of DA (100 µM, orange bar), as indicated; bar graph, statistical summary (mean ± SEM over the experimental epochs); *P < 0.05, significance level relative to baseline (dashed line). **C**: Plot, average time course of intracellular [Ca^2+^] (mean ± SEM, *n* = 17) during the application of a receptor blocker cocktail (in µM: 1 MPEP, 50 PTX, 1 TTX, 1 CGP52432, 50 A‐AP5, 25 DNQX, 100 LY367385, 0.5 LY341495) followed by DA application (100 µM, orange bar), as indicated; bar graph, statistical summary (mean ± SEM over the experimental epochs); *P < 0.05, **P < 0.01, ***P < 0.005, significance level relative to baseline [Ca^2+^] (dashed line). **D**: Plot, average time course of intracellular [Ca^2+^] (mean ± SEM, *n* = 11) during the application of the D1/5 receptor blocker SCH23390 (20 μM) followed by DA application (100 µM, orange bar), as indicated; bar graph, statistical summary; notation as in B. **E**: Plot, average time course of Ca^2+^‐dependent fluorescence (*ΔF/F* of Fluo‐4; mean ± SEM, *n* = 5) in GJC astroglia (hollow circles) and in peripheral processes of PAC (PAC‐p, filled circles) during the application of the L‐type Ca^2+^ channel blocker nifedipine (20 μM) followed by DA application (100 µM, orange bar), as indicated; bar graph, statistical summary (*ΔF/F* mean ± SEM over the experimental epochs); PAC‐p data are marked by light grey borders; other notations as in B–D.

The Fluo‐4 fluorescence intensity measurements suggested that DA could induce both elevations and (subsequent) decreases in astroglial [Ca^2+^], in a DA receptor‐dependent manner (Fig. 1I). To validate and explore this further, we monitored internal [Ca^2+^] using a direct concentration readout based on the fluorescence life‐time imaging (FLIM) of OGB‐1, which is highly sensitive to free nanomolar Ca^2+^ but not to the dye concentration, photobleaching, or light scattering and absorption in tissue (Zheng et al., 2015). The OGB‐1 FLIM measure could clearly detect a DA‐induced elevation followed by a decrease in [Ca^2+^] (Fig. 3A,B). A powerful cocktail of ionotropic and metabotropic receptor‐channel blockers applied to eliminate network influences left this [Ca^2+^] elevation largely unaffected (Fig. 3C). Intriguingly, the FLIM approach could also detect a relatively small [Ca^2+^] decrease upon the cocktail application alone (Fig. 3C), consistent with some contribution of neuronal activity to basal astroglial [Ca^2+^] (Di Castro et al., 2011; Panatier et al., 2011).

In line with the Fluo‐4 fluorescence intensity data (Fig. 1I), these experiments also showed that DA application under the blockade of D1/5 receptor with SCH23390 lowered basal [Ca^2+^] (Fig. 3D; FLIM also detected a slight [Ca^2+^] decrease upon SCH23390 application alone). It was earlier suggested that D2/3 receptor activation can reduce Ca^2+^ mobilization through modulation of L‐type VGCCs in rat *nucleus accumbens* neurons of the rat (Perez et al., 2011); the involvement of voltage‐gated Ca^2+^ channels would also seem consistent with the occlusion of DA‐induced [Ca^2+^] decreases through the suppression of network influences on astroglia (Fig. 3C). Intriguingly, we found that an L‐type Ca^2+^ channel blocker nifedipine did reduce basal [Ca^2+^], which occluded any subsequent DA‐induced [Ca^2+^] decreases while leaving [Ca^2+^] elevations qualitatively intact, but only in the processes of the patched cell (Fig. 3E). The somata of GJC astroglia remained non‐responsive to nifedipine (Fig. 3E; fluorescence signal in the processes was too weak for reliable assessment), thus suggesting the cell‐compartment specific sensitivity to Ca^2+^ channel blockade.

### DA Induces High‐Threshold Ca^2+^‐Elevations in Perforant Path Astroglia While Independently Inhibiting Local Synapses

**Figure 4:**
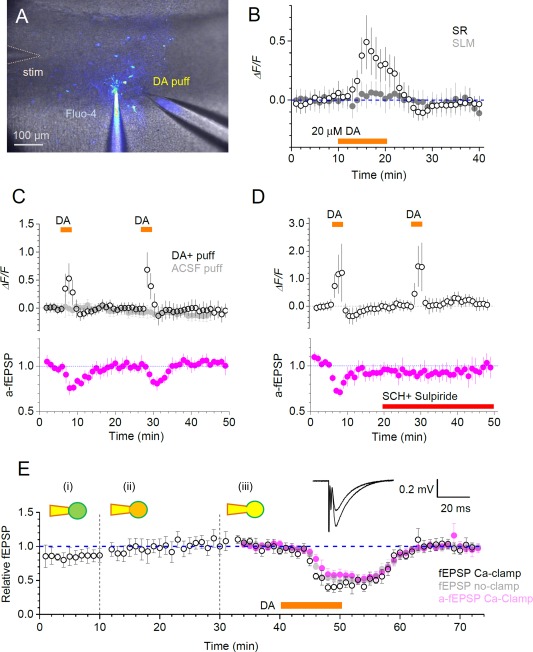
Putative roles of astrocytes in hippocampal circuit dopamine signalling. **A**: Experimental arrangement in *s. lacunose moleculare* (SLM), showing recording whole‐cell pipette (Fluo‐4), dopamine pressure application pipette (DA puff), and stimulating electrode (stim) shown; patched astroglia and GJCs stained via gap‐junction escape of Fluo‐4 can be seen. **B**: Distinct dopamine (DA) sensitivity of astroglia in *s. radiatum* (SR) and SLM: Time‐course (mean ± SEM) of Ca^2+^ response to the bath application of 20 μM DA in SR (hollow) and SLM (filled) GJCs, *n* = 12 and *n* = 10, respectively; difference is at least at *P* < 0.05 (unpaired *t*‐test) over the 15‐20 min window. **C**: Open circles, time course (mean ± SEM) of Ca^2+^ ‐dependent (Fluo‐4) fluorescence of GJCs in SLM during two brief (3 min) local DA puffs (200 µM in the puff pipette, as in A), as shown (*n* = 10); grey filled circles, control experiments with no DA in the puff pipette (*n* = 7); magenta, time course of a‐fEPSPs recorded from the patched astroglia (*n* = 3) indicating reversible dopaminergic inhibition of perforant path—CA1 synapses, reported earlier. **D**: Experiments as in C but with DA receptor blockers 5 µM SCH23390 and 20 µM Sulpiride applied after the first stimulus (red) abolishing synaptic inhibition (bottom, *n* = 3) but not astroglial Ca^2+^ responses (top, *n* = 6); notations as in C. **E**: Dopaminergic inhibition of perforant path fEPSPs or a‐fEPSPs persists after astrocytic Ca^2+^ clamp. Open circles, time course (mean ± SEM) of normalized fEPSP slope for cell‐attached (i), whole‐cell equilibrating (ii) and whole‐cell recording (iii) followed by bath application of DA (black bar) (*n* = 4); magenta, a‐fEPSPs recorded form the patched cell in Ca‐clamp conditions (*n* = 4); grey filled circles, control fEPSPs, with no Ca‐clamp (*n* = 6); traces, examples of fEPSPs of varied amplitudes recorded in one experiment. [Color figure can be viewed at wileyonlinelibrary.com]

To understand the physiological implications of prominent DA‐induced Ca^2+^ signals in astroglia we asked if such signaling could play a role in regulation of local synaptic transmission. It was previously shown that in hippocampal area CA1 DA application inhibits excitatory synaptic transmission, in particular at perforant path‐CA1 synapses in *stratum lacunosum moleculare* (SLM, at concentrations of 20 µM and higher) (Otmakhova and Lisman, 1999). We therefore asked whether this phenomenon could be explained, at least partly, by the DA‐induced Ca^2+^‐dependent activity of local astroglia. To restrict our experimental manipulations to the SLM region of CA1 pyramidal cell apical dendrites, we applied DA through a patch pipette located in the vicinity of the patched SLM astrocyte (Fig. 4A). First, however, we found that bath application of 20 µM DA, while inducing a clear [Ca^2+^] increase in GJC astroglia in the *stratum radiatum* (Fig. 1F), had no effect on [Ca^2+^] in SLM astroglia (Fig. 4A). Only when DA concentration was increased to 200 µM in the application pipette (Fig. 4A), its application (in paired‐stimulus tests, to minimize rundown and false‐negatives) induced a clear [Ca^2+^] rise (Fig. 4C; note the control for mechanical effects of puffing).

In full accord with the previously published observations, in these tests DA application also inhibited local a‐fEPSPs recorded through the astrocyte patch pipette: this method enabled us to deal with synapses adjacent to the patched astrocyte, or within the tissue domain occupied by the patched astrocyte (Henneberger and Rusakov, 2012). Strikingly, blocking DA receptors before the second DA stimulus completely abolished synaptic inhibition, which was consistent with the previous work (Otmakhova and Lisman, 1999), while—surprisingly—leaving astroglial [Ca^2+^] elevations intact (Fig. 4D).

To further test whether astrocyte Ca^2+^ activity is involved in DA‐induce synaptic inhibition in this circuitry, we held a local astrocyte first in cell‐attached mode, then in whole‐cell loading it with a Ca^2+^ clamping solution (to suppress internal Ca^2+^ signaling (Henneberger et al., 2010)) while recording perforant path stimulation‐induced local fEPSPs, before and during DA application. Monitoring synaptic activity, either with a local extracellular electrode near the patched astrocyte or, again, directly through the astrocyte pipette (Henneberger and Rusakov, 2012), showed no effect of Ca^2+^ clamp on DA‐induced synaptic inhibition (Fig. 4E). Thus, Ca^2+^ signals in SLM astroglia do not appear causally related to the DA‐induced inhibition of local perforant path‐CA1 pyramidal cell synapses.

## Discussion

Here we report that DA can trigger increases and decreases in intracellular astrocytic calcium concentration in acute brain slices. Astrocytes *in situ* are inter‐connected by gap junctions (Giaume and Theis, 2010), which are permeable to the fluorescence indicators we used (Fluo‐4, Alexa 594, OGB‐1). The diffusion of these dyes into neighboring astroglia allowed us to image Ca^2+^ signals in the GJC cells unperturbed by patch‐pipette dialysis (Zheng et al., 2015). As expected, the patched cell soma showed little Ca^2+^ sensitivity: it is a relatively small cell compartment (8–10 μM diameter) which will dialyze rapidly with the pipette solution, probably washing out small endogenous signaling molecules that could be vital for DA‐induced effects.

We found large DA‐induced increases in astrocytic [Ca^2+^], which were consistent with the previous findings in cultured astroglia (Parpura and Haydon, 2000; Requardt et al., 2012). Our tests showed that in *stratum radiatum* astroglia these increases were suppressed by the IP_3_ receptor blocker 2‐APB and occluded by Ca^2+^ released from internal stores or as a result of Ca^2+^ pump blockade. This is in line with the reported observations in culture suggesting that both D1‐type and D2‐type receptors trigger release of IP_3_ (Hasbi et al., 2009; Hernandez‐Lopez et al., 2000). In astrocytes, both the D1‐type receptor‐mediated activation of the PLC/IP_3_ pathway reported earlier (Liu et al., 2009c) and the direct effect of NADH increase on IP_3_ receptors, also mediated by D1 receptors (Requardt et al., 2012), could be responsible for this IP_3_‐dependent calcium increase. Intriguingly, DA‐induced Ca^2+^ elevations were not affected by intracellular IP_3_ buffering in the IP_3_‐sponge animals even though in these animals the classical mGluR‐induced Ca^2+^ waves had been found partially inhibited in CA1 astroglia (Tanaka et al., 2013). This observation suggests little role of IP_3_ diffusion over any appreciable distances in the phenomenon pointing instead to a nanodomain‐type interaction between IP_3_ and its receptor.

The ability of DA to *decrease* astrocytic Ca^2+^ was a novel and unexpected finding. In astrocytes *in situ*, previous evidence associated inducible Ca^2+^‐decreases with TRPA1 channel‐dependent regulation of basal [Ca^2+^] which was decreased by a specific blockade of these channels (Shigetomi et al., 2013). Interestingly, in peripheral neurons, apomorphine—a non‐selective dopamine receptor agonist with a slight preference toward D2‐type receptor activation—triggered concentration‐dependent activation (low concentrations) or inhibition (higher concentrations) of the TRPA1 channel (Schulze et al., 2013) (although see (Aman et al., 2007)). This novel site of potential dopamine action could be another explanation for the Ca^2+^‐decrease reported here. We also found that the blockade of D2/3 receptor, or both D1 and D2‐type receptors, inhibits DA‐induced Ca^2+^ response in either direction whereas blocking D1/5 receptor alone diminishes [Ca^2+^] increases (consistent with previous studies (Liu et al., 2009a; Requardt et al., 2012)) while leaving Ca^2+^ decreases unaffected. This suggests that Ca^2+^ increases are under the prevalent control of both D1 and D2‐type receptors (Hasbi et al., 2010; Hasbi et al., 2011), whereas Ca^2+^ decreases are only controlled by D2/3 receptors.

As for the D2/3 receptor‐mediated Ca^2+^ decrease, there is evidence for this function of D2‐type receptors in the literature: apomorphine (see above) can decrease Ca^2+^ in chromaffin cells through PLC inhibition (a recognized downstream signaling molecule of D2/3Rs) and Ca^2+^ entry into the cell (Sontag et al., 1990). Interestingly, in nerve cells of *nucleus accumbens*, D2/3 receptor activation has been shown to decrease calcium mobilization through modulation of L‐type VGCCs in rat (Perez et al., 2011), and in rodent prefrontal cortical neurons D4 receptors decrease NMDAR mediated Ca^2+^ currents (Wang et al., 2003). Thus, some studies suggest that D2/3R‐mediated decreases in intracellular Ca^2+^ could involve inhibition of Ca^2+^ channel activity. Correspondingly, astroglial Ca^2+^ concentration tends to decrease when extracellular Ca^2+^ is depleted (Verkhratsky and Parpura, 2014). As astrocytes are known to express functional VGCCs (Barres et al., 1989; Burgos et al., 2007; D'Ascenzo et al., 2004; Padmashri and Sikdar, 2007), these channels could therefore underlie the mechanism of DA‐induced Ca^2+^ decreases. Indeed, in our experiments an L‐type Ca^2+^ channel blocker nifedipine occluded such decreases—albeit in astrocyte processes only. Whilst this suggests a role for the network‐activity dependent Ca^2+^ channel entry in maintaining astroglial [Ca^2+^], the relationship between somatic and peripheral mechanisms of Ca^2+^ control requires further studies. Interestingly, the level of oxidative stress in cultured neurons has been found to control the effect of dopamine on calcium levels, with higher oxidative stress (such as may be found in the presence of high concentrations of dopamine, see (Vaarmann et al., 2010)) triggering D2/3R‐dependent inhibition of local VGCC opening (Steullet et al., 2008), although this has also been reported to trigger TRP channel opening (Kim and Hwang, 2013).

Unlike the prominent Ca^2+^ rises recorded from astrocytes in *stratum radiatum* in response to a 20 µM DA application, SLM astrocytes showed Ca^2+^ increases only when a 200 μM DA stimulus was applied. At these relatively high concentrations, some of the DA applied in SLM could have triggered Ca^2+^ responses in the more sensitive *stratum radiatum* astrocytes nearby. Nonetheless, Ca^2+^ responses of SLM astroglia were DA receptor independent and therefore could not have resulted from Ca^2+^ signal propagating from *stratum radiatum* astrocytes in which [Ca^2+^] elevations were DA receptor dependent. This observation indicates a clear difference in sensitivity to DA between astrocytes from different hippocampal strata. Some morphological differences between *stratum radiatum* and SLM astrocytes have been reported before, SLM astrocytes occupying smaller synaptic territories (Ogata and Kosaka, 2002). Similarly, astrocytic K^+^ buffering in SLM has been reported to be more gap‐junction dependent than in the *stratum radiatum* (Hewett, 2009; Wallraff et al., 2006). This hippocampal‐region sensitivity to DA appears inversed when applied to local neural circuits: DA application prominently inhibits perforant path synapses on CA1 pyramidal cells (in SLM) while having little effect (one which inversely depends on DA concentration) on Schaffer collateral synapses (in *stratum radiatum*) (Otmakhova and Lisman, 1999). Indeed, we found that blockade of DA receptors had no effect on DA‐induced Ca^2+^ rises in SLM astroglia while eliminating the DA‐induced local synaptic inhibition, whereas the latter was unaffected by Ca^2+^ clamp in SLM astroglia. Interestingly, the DA receptor independence of DA‐induced Ca^2+^ rises in SLM astrocytes resembles that in cultured astroglia (Vaarmann et al., 2010), pointing to the fact that DA effects are not universal among astrocytes in different brain regions or across preparations. This result also strongly suggests that the DA signaling pathways acting on synapses and astroglia in SLM are not causally related. Thus, unlike other common neurotransmitters and neuromodulators (such as glutamate, GABA, ATP, or adenosine) dopamine signaling could, at least in some cases, engage astroglia and local neural circuits independently. The adaptive role of this mode of action remains to be ascertained: clearly, this DA target divergence and the cellular mechanisms involved require a dedicated study. In a wider context, our findings demonstrate that prominent Ca^2+^ elevations in astroglia, which are often associated with significant effects on local synaptic function may be consequential to multiple and diverse cellular cascades which do not necessarily lead to similar physiological consequences (Bazargani and Attwell, 2016; Khakh and Sofroniew, 2015; Rusakov, 2015; Volterra et al., 2014).

## Author Contributions

A.J. planned and carried out experiments and data analyses; O.T. and K.Z. designed and carried out FLIM experiments; D.A.R, L.B. and C.H. contributed to experiment planning, selected experiments and data analyses; A.S. contributed to experiments and analyses in IP_3_‐sponge mice; D.A.R. and C.H. narrated the study, and A.J. and D.A.R. wrote the paper.
